# Isoluminant stimuli in a familiar discrete keying sequence task can be ignored

**DOI:** 10.1007/s00426-019-01277-0

**Published:** 2019-12-06

**Authors:** Willem B. Verwey

**Affiliations:** 1 Faculty of Behavioral Sciences, Cognitive Psychology and Ergonomics, University of Twente, P.O. Box 217, 7500 AE Enschede, The Netherlands; 2 Human Performance Laboratories, Department of Health and Kinesiology, Texas A&M University, College Station, TX USA

## Abstract

Motor sequencing models suggest that when with extensive practice sequence representations have developed, stimuli indicating the individual sequence elements may no longer be used for sequence execution. However, it is not clear whether participants can at all refrain from processing these stimuli. Two experiments were performed in which participants practiced two 7-keypress sequences by responding to isoluminant key-specific stimuli. In the mixed condition of the ensuing test phase, the stimuli were displayed only occasionally, and the question was whether this would make participants stop processing these stimuli. In Experiment 1, the benefit of displaying stimuli was assessed after substantial practice, while Experiment 2 examined development of this benefit across practice. The results of Experiment 1 showed that participants rely a little less on these stimuli when they are displayed only occasionally, but Experiment 2 revealed that participants quickly developed high awareness, and that they ignored these stimuli already after limited practice. These findings confirm that participants can choose to ignore these isoluminant stimuli but tend to use them when they are displayed. These and other findings show in some detail how various cognitive systems interact to produce familiar keying sequences.

## Introduction

The development of sequential movement skills is investigated with a variety of experimental procedures (for reviews, see, e.g., Abrahamse, Jiménez, Verwey, & Clegg, 2010; Doyon et al., 2009; Perruchet & Pacton, 2006; Rhodes, Bullock, Verwey, Averbeck, & Page, 2004; Rosenbaum, 2010; Verwey, Shea, & Wright, 2015). One of these procedures involves participants initially reacting to each of two fixed series of 2–7 successively presented key-specific stimuli in the so-called *discrete sequence production* (DSP) task (Abrahamse, Ruitenberg, De Kleine, & Verwey, 2013; Verwey, 1999). With practice, participants usually can perform the two sequences in response to just the first key-specific stimulus. This suggests that eventually they may ignore the stimuli after the first one. Still, there are reasons to assume that, if displayed, the use of key-specific stimuli may be mandatory. The present study therefore addressed whether participants stop processing key-specific stimuli when they are displayed only occasionally. I used isoluminant color changes as stimuli to explore this for the situation that stimulus display attracts little or no attention.

### Developing motor sequencing skill

When participants practice discrete keying sequences, they begin by reacting to individual key-specific stimuli. However, the *Cognitive framework for Sequential Motor Behavior* (C-SMB) posits that already within tens of trials participants develop spatial and/or verbal central-symbolic sequence representations (Barnhoorn, Döhring, Van Asseldonk, & Verwey, 2016; Verwey, 2015; Verwey et al., 2015; for support from brain imaging studies, see Hikosaka et al., 1999; Verwey et al., 2019). Extracting individual responses from these spatial and verbal representations demands central-cognitive processing resources, and this makes sequence execution susceptible to interference by other cognitively loading tasks (Verwey, Abrahamse, & De Kleine, 2010; Verwey, Abrahamse, De Kleine, & Ruitenberg, 2014).

After hundreds of trials, sequence representations develop in terms of motor parameters like activation patterns of agonist/antagonist muscles (Shea, Kovacs, & Panzer, 2011), musculoskeletal forces and dynamics (Krakauer, Ghilardi, & Ghez, 1999), joint angles (Criscimagna-Hemminger, Donchin, Gazzaniga, & Shadmehr, 2003), and/or posture-related representations (Rosenbaum et al., 2009). These representations are denoted *motor chunks* (Broadbent, 1987; Graybiel, 1998; Sakai, Hikosaka, & Nakamura, 2004; Verwey, 1996). The use of motor chunks is characterized by effector-specific sequence learning (Verwey & Wright, 2004) and an overlap between successive movements (i.e., coarticulation; see, e.g., Gentner, Grudin, & Conway, 1980; Gonzalez-Sanchez, Dahl, Hatfield, & Godøy, 2019). Executing motor sequences based on motor chunks is fast because these representations code the sequences motorically and executing the individual responses demands few central-cognitive processing resources. The required cognitive processes merely involve preparing, selecting, and initiating motor chunks and no longer deriving response codes from sequence representations.

Research demonstrated that when discrete keying sequences exceed about 4 or 5 responses, usually a relatively slow response develops that divides the sequence in segments of about 3 or 4 responses (Acuna et al., 2014; Verwey, 1999; Verwey & Eikelboom, 2003; Wymbs, Bassett, Mucha, Porter, & Grafton, 2012). These slow responses suggest that some dominant—central-symbolic or motor chunk—representation has a limited capacity and the slow response indicates the transition from one to the next sequence representation. The first response of the second and later segments is called a *concatenation response*; the other responses past the first one are execution responses (Abrahamse et al., 2013).

According to C-SMB, the systems responsible for reacting to stimuli and for applying the central-symbolic and motor chunk representations are functionally separate systems that race to trigger each next response (Verwey, 2003; for other racing cognitive systems see Brown & Heathcote, 2008; Raab, 1962; Ratcliff, 2006; Ulrich & Miller, 1997). As these systems are stochastic and provide their output at times distributed around some average, a generally slower system may increase general execution rate because it occasionally still wins the race (Verwey, 2003).

### The contribution of key-specific stimuli

The development of sequence representations in a DSP task suggests that the contribution of the second and later key-specific stimuli reduces with practice. Indeed, no longer displaying key-specific stimuli in a study with DSP sequences slowed individual responses by 155 ms after 144 practice trials per sequence (Verwey, Abrahamse, Ruitenberg, Jiménez, & De Kleine, 2011), and by only 32 ms after 720 practice trials (Ruitenberg, Verwey, Schutter, & Abrahamse, 2014). Sequencing models suggest that with even more practice the stimulus–response (S–R) translation system may be entirely outrun by the sequencing systems (Abrahamse et al., 2013; Verwey et al., 2015). Participants could then refrain from processing these stimuli. In line with this idea, fully aware participants in a serial RT task study involving an 8-element binary sequence ignored stimuli already after 90 sequence repetitions (Tubau & López-Moliner, 2004). Earlier DSP studies did not show this independence from key-specific stimuli (Ruitenberg et al., 2014; Verwey, 1999; Verwey et al., 2011), but participants in DSP task studies are usually not fully aware of their sequences (as reviewed in Verwey, Groen, & Wright, 2016). Perhaps participants refrain from using key-specific stimuli only when they have full sequence knowledge, or know they have.

Nevertheless, it is possible also that key-specific stimuli continue to contribute because participants cannot easily ignore them, and that the results reported by Tubau and López-Moliner (2004) will not be found with the DSP task. This is suggested by studies of visual search and response priming. That is, the luminance change associated with stimulus display is known to automatically capture visuospatial attention (Jonides & Yantis, 1988; Yantis & Jonides, 1984), and the resulting directing of this attention then primes the spatially compatible response (Rizzolatti, Riggio, Dascola, & Umiltá, 1987; Van der Lubbe, Abrahamse, & De Kleine, 2012). In the serial RT task reported by Tubau and López-Moliner (2004), aware participants may have been able to disregard the key-specific stimuli because they entirely disengaged attention from the area where the stimuli were displayed (Belopolsky, Schreij, & Theeuwes, 2010; Theeuwes, 2010). This may not be possible in the DSP task in which participants always need to identify the first stimulus. So, while models of sequencing tasks suggest that participants may eventually ignore the non-initial key-specific stimuli in discrete keying sequences, models of attentional capture and response priming suggest that when in the DSP task key-specific stimuli are displayed participants will not be able to ignore them.

### The present experiments

The purpose of the present study was to explore whether participants will always process key-specific stimuli or whether they may eventually be able to stop processing them. Below, we refer to stimulus processing and response priming as opening the *S–R translation channel*. This channel operates in parallel with the central-symbolic and motor chunk sequencing systems to trigger individual responses. I distinguished three hypotheses. In short, these state that (a) after practice key-specific stimuli are no longer used, (b) participants can control whether these stimuli are being used, and (c) stimuli processing cannot be intentionally controlled and the stimuli continue to be used regardless of the amount of practice.

More specifically, the *fast-sequencing hypothesis* posits that with practice sequencing systems become so fast triggering individual responses that the contribution of S–R translation is eventually outrun. The S–R channel may remain open, but this no longer influences execution rate (Verwey, 2003). This hypothesis is in line with the smaller benefit after 144 practice trials than after 720 practice trials (Ruitenberg et al., 2014; Verwey et al., 2011), and predicts that the benefit of stimulus display in the DSP task will vanish with even more extensive practice.

It is possible also that sequencing systems never become fast enough to outrun S–R translation. In that case, the *intentional S–R translation hypothesis* posits that participants can purposely open and close the S–R channel. For example, participants may close the S–R channel because keeping it open is not worth the cognitive effort. This may entail participants attending to, or disengaging attention from, the stimulus display area (Belopolsky et al., 2010; Theeuwes, 2010). They may choose to stop processing key-specific stimuli because they know they have full sequence awareness (cf. Tubau & López-Moliner, 2004), or because they realize that stimulus processing has little merit. Conversely, participants may choose to continue processing key-specific stimuli because that takes little effort.

Finally, the *mandatory processing hypothesis* posits that the S–R channel remains open and key-specific stimuli in the DSP task will always race to trigger the spatially compatible response. Lasting stimulus processing might happen because the first stimulus needs to be identified in the DSP task and participants cannot quickly disengage attention from the stimulus display area, for example, because of the cognitive demands of sequence execution.

The intentional S–R translation hypothesis differs from the fast-sequencing and the mandatory processing hypotheses in that only the former predicts that the benefit of individual key-specific stimuli varies with details of the task, like sequence awareness and stimulus display frequency. We tested these three hypotheses in two experiments. Both started off with participants practicing two 7-key DSP sequences. They then executed the practiced sequences in three test conditions. In the most important mixed RSI condition, there was a 20 % chance that the key-specific stimulus was displayed immediately. Otherwise that stimulus appeared only when no response had been given within 800 ms. In Experiment 1, the main research question was whether after extended practice this almost unexpected display of a key-specific stimulus would still benefit the ensuing response. That would show that the S–R channel had remained open. As the results indicated partial opening of the S–R channel, Experiment 2 then explored whether the contribution of the occasionally displayed stimuli would reduce across practice, as suggested by the fast-sequencing hypothesis. Awareness was assessed to see whether that would be associated with the benefit of stimulus display. I investigated this with isoluminant stimuli to determine whether the S–R channel can at least be controlled for the situation that key-specific stimuli do not capture attention (Jonides & Yantis, 1988; Theeuwes, 1995).

The present paradigm allowed also a test of C-SMB’s race assumption. When key-specific stimuli are still being used for sequence execution, the race assumption posits that their contribution to sequence execution should be larger for the slow concatenation responses than for the fast execution responses. This prediction follows from the assumption that the concatenation response is triggered more slowly by sequence representations than the other responses, so that reacting to key-specific stimuli should win the race more often for the concatenation than for the other non-initial responses.

## Experiment 1

After 450 practices trials per sequence, participants performed in a test phase with three conditions. The *fixed 0-RSI condition* entailed the normal DSP task procedure with the 0 RSIs that had been used also during practice. In the *fixed 800-RSI condition*, the stimuli, past the first one, were displayed only when no response had been given within 800 ms. As the participants had been instructed to try pressing the required key without waiting for the stimuli, the entire familiar sequences could be executed in response to just the first stimulus. This would mimic the so-called single-stimulus condition of earlier DSP studies (Ruitenberg et al., 2014; Verwey, 1999; Verwey et al., 2011), except that this time participants knew that the key-specific stimulus would appear if they would not respond within 800 ms. Like in various earlier studies (Barnhoorn, Panzer, Godde, & Verwey, 2019; Ruitenberg et al., 2014; Verwey, 1999; Verwey et al., 2011), RTs were expected to be shorter when stimuli were displayed in the fixed 0-RSI condition than when they were not in the fixed 800-RSI condition.

The third test condition was the important *mixed RSI condition*. It included a mix of the fixed 800-RSI and the fixed 0-RSI conditions. For each non-initial response in the sequence, there was a 20 % probability that the key-specific stimulus was displayed immediately after the preceding response. This was the *mixed 0-RSI condition*. The remaining 80 % of the non-initial responses involved the *mixed 800-RSI condition* in which the stimulus was presented only when after 800 ms no response had yet been given. If in the mixed conditions the S–R channel would be closed, responses in the mixed 0-RSI should be as fast as those in the mixed 800-RSI conditions and slower than in the fixed 0-RSI condition. If the S–R channel would be open in the mixed RSI condition, responses in the mixed 0-RSI should be faster than in the mixed 800-RSI conditions and about as fast as responses in the fixed 0-RSI condition.

I tested the prediction of the race assumption that the beneficial effect of stimulus display would be larger for concatenation than for execution responses, with sequences in which concatenation was imposed (so-called *prestructured sequences*), and with sequences in which concatenation is known to develop spontaneously (i.e., *unstructured sequences*). I looked at these two types of sequences because earlier studies had indicated that imposed and spontaneous concatenation may involve different processes. This was suggested by the different effects of task context on the concatenation of successive segments in unstructured and prestructured sequences (Ruitenberg, Verwey, & Abrahamse, 2015). Also, an imaging study with the DSP task suggested that cognitive load of concatenation was higher when a pause had been removed from prestructured sequences than in unstructured DSP sequences in that activity was higher in typical executive areas of the brain (i.e., the dorsolateral prefrontal cortex and the premotor cortex, Jouen et al., 2013). A higher cognitive load may reflect a cognitively demanding concatenation process. This concatenation process could therefore be slowed by processing of the occasional early stimulus in prestructured sequences, while concatenation in the unstructured sequence would not be slowed. In that case, the predicted relatively large RT benefit of early stimulus display on the concatenation response would be smaller in the prestructured than in the unstructured sequences.

Unstructured sequences exceeding 4 or 5 responses usually show one or more relatively slow responses that are attributed to concatenation (Acuna et al., 2014; Verwey, Abrahamse, & Jiménez, 2009; Verwey & Eikelboom, 2003). This may occur at individually different sequence positions (Acuna et al., 2014; Verwey et al., 2009; Verwey & Eikelboom, 2003), but I used sequences that typically show a relatively slow fifth response (i.e., R_5_) across all participants (De Kleine & Verwey, 2009; Ruitenberg, De Kleine, Van der Lubbe, Verwey, & Abrahamse, 2012; Verwey et al., 2014). The prestructured sequences were practiced with a short pause that preceded the display of the fifth stimulus (i.e., S_5_). When in the ensuing test phase this pause is removed R_5_ is usually quite slow. This would reflect concatenation at that position for all participants (Verwey, 1996; Verwey et al., 2014; Verwey et al., 2009; Verwey & Dronkert, 1996). This effect of the pause may well be due to the longer time between successive responses during practice reducing the development of response–response associations (Verwey & Dronkers, 2019).

### Method

#### Participants

Forty-eight undergraduate students took part in Experiment 1 (age range 18–26, plus one 44 years old, average 20.9, 27 women, 11 lefthanders) in exchange for course credits. As the effect size in this particular study was difficult to estimate from earlier studies, I figured that the experiment should be able to detect medium effect sizes, that is, $\eta_{\text{p}}^{ 2}$ = 0.06. Together with the more typical parameters in power analyses (*α *= 0.05, power 1−*β *= 0.85), GPower computed for the within factors of a repeated measures ANOVA a sample size of 38 participants. I rounded this up to 48 in order to reach a multiple of 12 that was needed to fully balance across four finger positions per sequence element and three test conditions. Three participants were replaced because they had misunderstood the instruction and had always waited for the stimulus to be displayed. The participants reported normal vision or had vision correction. None was color blind (see below). Every participant signed an informed consent at the beginning of the experiment. The study had been approved by the ethics committee of Faculty of Behavioral Sciences of the University of Twente.

#### Apparatus

Stimulus presentation, timing, and data collection were achieved using the E-prime© 2.0 experimental software package on a standard Windows 7 PC. Unnecessary Windows services were shut down to improve RT measurement accuracy. Stimuli were presented on a 17 inch Philips 107T5 CRT display running at 1024 by 768 pixel resolution in 32 bit color, and refreshing at 85 Hz. The viewing distance was approximately 50 cm, but this was not strictly controlled.

#### Task

The task involved presentation of four 0.9 × 0.9 cm square placeholders horizontally in the center of the computer screen against a gray background. The placeholders consisted of black lines with a gray filling as default. There were 0.7 cm gaps between the four placeholders. Participants sat with their left-hand fingers resting lightly on the C V B and N keys of a regular computer keyboard. The imperative stimulus, indicating that the corresponding key was to be pressed, consisted of a color change in that the default gray placeholder color was filled with light blue. The gray background and the blue placeholder filling had the same luminance (28.1 cd/m^2^, as tested with a Macam L203 photometer with a CIE FOV-101 luminance probe) to assure that RT effects could not be attributed to the arousal induced by onset of a bright stimulus (Kahneman, 1973; Luce, 1986; Piéron, 1920), or to attention attraction (Jonides & Yantis, 1988; Yantis & Jonides, 1984).

When the correct key had been pressed, the color in the square changed back to the gray background color. The participants could release the key whenever they wanted. Errors resulted in the message “wrong key” for 2000 ms after which the sequence was broken off. The message “too early” was displayed for 500 ms when participants pressed a key before presentation of the first stimulus of a sequence, or before the stimulus following the pause in the prestructured sequence during practice.

Stimuli were presented in two fixed series of seven (i.e., S_1_–S_7_), thus requiring two fixed sequences of seven key presses (R_1_–R_7_). Below, combinations of responses are indicated by combining indices, like R_23467_ indicating the combinations of the responses at Positions 2, 3, 4, 6, and 7 (i.e., R_2_, R_3_, R_4_, R_6_, and R_7_). The term trial is used to denote an entire sequence, and the two 7-key sequences were always presented in random order. The time between stimulus *n* and response *n* is indicated by *T*_*n*_ and signifies the response time. In case of response–stimulus interval (RSI) 0, this response time equals the interkey interval (e.g., the response time between S_2_ and R_2_ is T_2_). Each sequence was followed by blanking the display for 2 s and then displaying the empty placeholders again for 500 ms. Then, S_1_ was displayed again.

The two sequences of each participant were selected from a set of four versions and, across participants, each sequence was used as often as prestructured and unstructured sequences. The four sequences were created by mapping the numbers of the series 1323124 to each of the four keys so that, across participants, each finger occurred as often at a particular sequential location. For example, one participant had VNBNVBC and NVCV-NCB (‘-‘indicating the pause in the practice phase), the next participant had CBVBCVN and BCNC-BNV, and so on.

#### The practice phase

The practice phase involved five practice blocks, each including 90 unstructured and 90 prestructured sequences, yielding a total of 450 practice trials for each sequence. Each practice block lasted 10–15 min and was followed by a 4-min rest period. Halfway through each practice block there was a 40-s break.

During practice, the prestructured sequences included a pause between R_4_ and S_5_ to impose a segmentation structure (e.g., Verwey & Dronkers, 2019; Verwey & Dronkert, 1996). As fixed RSIs could induce learning of a particular rhythm, and therewith perhaps prevent development of two motor chunks, I used a non-aging interval^1^. These pauses in prestructured sequences are known to induce a clear boundary between two successive motor chunks (Verwey, 1996). The unstructured sequence did not contain this pause, but I used sequences that have repeatedly been shown to spontaneously induce a relatively slow R_5_ (the same orders as in De Kleine & Verwey, 2009; Ruitenberg et al., 2012; Verwey et al., 2014).

#### The test phase

The 6th and last block included the test phase. It consisted of three subblocks with 40 unstructured and 40 prestructured sequences administered in a random order. The pause no longer occurred during the prestructured sequences. Before commencing, participants read an instruction screen explaining the three conditions. The order of the three subblocks was counterbalanced across the participants, and these were separated each time only by a brief instruction for the oncoming task.

In the fixed 0-RSI condition, S_2_–S_7_ were presented immediately after the preceding key press (RSI 0). In the fixed 800-RSI condition, the stimulus was presented after an RSI of 800 ms unless the correct key had been pressed earlier. In that case the participants could immediately press the next key without waiting. The instruction encouraged participants to press each next key without waiting for the appearance of the key-specific stimulus. Finally, in the mixed RSI condition, there was an 80 % likelihood that presentation of key-specific stimuli at Positions 2 to 7 (i.e., S_2_–S_7_) occurred only when after 800 ms no response had yet been pressed. Only in the remaining 20 % of the responses, the stimulus was displayed immediately after depressing the preceding response. As this was determined independently for each individual stimulus, a sequence in the mixed RSI condition could include no 0-RSIs, but also several 0-RSIs. Again, participants were encouraged to press each next key without waiting for the key-specific stimulus.

#### Procedure

Upon entering the laboratory, the participants filled out an informed consent form and received a written instruction on the task to be performed. If unclear, this instruction was orally extended by the experimenter. Given that the stimulus consisted of a color change, participants were tested for color blindness using a simplified version of the Ishihara test (e.g., Birch, 1997). Then, five practice blocks were carried out after which the participants filled out an awareness questionnaire. After informing them that there had been two 7-key sequences, it asked the participants to write down their sequences from memory in terms of the keys they had pressed. The relative location of these keys was depicted in the questionnaire. Finally, the participants executed the test phase in Block 6. The duration of the experiment was about two and a half hours.

### Results

#### Practice blocks

The mean RTs of errorless sequences in the practice phase were analyzed with a within-subjects 5 (Block) × 2 (Structure: sequence with/without pause before S_5_) × 7 (Key) ANOVA. The first two sequences in each subblock were excluded. This ANOVA showed main effects for Block, *F*(4,188) = 379.2, *p *< 0.001, $\eta_{\text{p}}^{ 2}$ = 0.89, and Key, *F*(6,282) = 178.9, *p *< 0.001, $\eta_{\text{p}}^{ 2}$ = 0.79, that according to visual inspection of the data resulted from decreasing reaction times with practice, and relatively slow key presses at R_1_ and R_5_. Visual inspection further showed that the Block × Key interaction reflected the typical phenomenon with these sequences that practice reduced T_23467_ more than T_15_, *F*(24,1128) = 46.9, *p *< 0.001, $\eta_{\text{p}}^{ 2}$ = 50. According to the Structure × Key interaction, the T_5_ versus T_23467_ difference was larger in the prestructured than in the unstructured sequence, *F*(6,282) = 9.9, *p *< 0.001, $\eta_{\text{p}}^{ 2}$= 0.17, and the Structure × Key × Block interaction indicated that this relative slowness of R_5_ in the prestructured sequence increased across practice, *F*(24,1128) = 6.3, *p *< 0.001, $\eta_{\text{p}}^{ 2}$= 0.12. This was to be expected in the practice phase with the time uncertainty induced by the non-aging interval preceding S_5_ in the prestructured sequence. However, planned comparisons showed that R_5_ was not only slower than R_23467_ in the prestructured but also in the unstructured sequences, *F*s(1,47) > 9.7, *p*s < 0.004, $\eta_{\text{p}}^{ 2}$s > 0.17.

According to an ANOVA with the same design on arcsine transformed error proportions (Winer, Brown, & Michels, 1991), error rate increased with practice block from 1.4 % per key in Block 1 to 2.0 % in Block 5, *F*(4,188) = 12.4, *p *< 0.001, $\eta_{\text{p}}^{ 2}$= 0.21. Furthermore, error rate varied with sequential position between 0.8 % (at R_1_) and 2.7 % (R_2_), *F*(6,282) = 23.1, *p *< 0.001, $\eta_{\text{p}}^{ 2}$= 0.33. The difference across sequential positions was larger for the prestructured than for the unstructured sequence, *F*(6,282) = 6.8, *p *< 0.001, $\eta_{\text{p}}^{ 2}$= 0.13, but always remained below 3 % per key.

#### Test blocks

A first analysis examined the proportion of responses over 800 ms (that were probably given to the key-specific stimuli). It involved a 2 (RSI: 0 vs. 800 ms) × 2 (FixMix: Fixed vs. Mixed RSI conditions) × 2 (Structure: sequence with/without pause before S_5_ during practice) × 6 (Key: R_234567_) repeated measures ANOVA on the arcsine transformed proportion of responses over 800 ms. Unsurprisingly, the RSI main effect showed that the proportion of the responses that took longer than 800 ms was larger in the 800-RSI condition than in the 0-RSI condition, 6.5 % vs. 1.0 %, *F*(1,46) = 35.1, *p *< 0.001, $\eta_{\text{p}}^{ 2}$= 0.43. The Key main effect, *F*(5,230) = 14.0, *p *< 0.001, $\eta_{\text{p}}^{ 2}$= 0.23, and the RSI × Key interaction, *F*(5,230) = 8.5, *p *< 0.001, $\eta_{\text{p}}^{ 2}$= 0.16, together showed that the proportion of slow, stimulus-dependent, responses was quite consistent across key positions in the 0-RSI condition (between 1.8 % and 0.5 %). In contrast, in the RSI 800 conditions, the number of stimulus-dependent responses was highest for R_2_ and R_4_ (8.5 % and 8.9 %, respectively) and was lower for R_3567_, especially for the last two responses (6.1 %, 7.3 %, 5.4 %, and 2.9 %, respectively). So, there was a monotonous decrease in stimulus-dependent responses between R_4_ and R_7_ from 8.9 %, 7.3 %, and 5.4 %, to 2.9 %, respectively.

RTs obtained in errorless sequences of the test block were analyzed with a repeated measures ANOVA with the same 2 (RSI) × 2 (FixMix) × 2 (Structure) × 6 (Key: R_2_–R_7_) design as mentioned above. Only sequences were included of which none of the responses after R_1_ had been given to a stimulus (i.e., with all RTs below 800 ms). This criterion excluded 3 %, 17 %, and 17 % of the sequences in the fixed 0-RSI, fixed 800-RSI, and mixed RSI conditions, respectively. This ANOVA showed main effects of RSI, *F*(1,47) = 33.3, *p *< 0.001, $\eta_{\text{p}}^{ 2}$= 0.41, and Key, *F*(5,235) = 41.2, *p *< 0.001, $\eta_{\text{p}}^{ 2}$= 0.47. These effects indicated that RTs were shorter in the 0-RSI than in the 800-RSI condition, and that after exclusion of R_1_ the Key effect could be especially attributed to a slow R_5_.

**Fig. 1:**
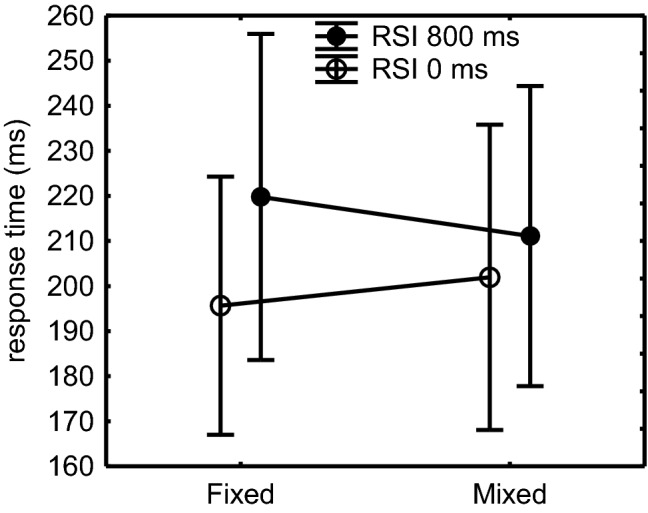
Effects in Experiment 1 of stimulus display on the averages of T_2_–T_7_ in the RSI 0 condition relative to the RSI 800 condition in the fixed and the mixed conditions of the test block. Sequences were included only when all T_2_–T_7_s were below 800 ms. Error bars indicate the standard error of the mean (SEM; these values are quite large because they also include the differences across T_2_–T_7_, cf. Fig. 2)

Significance of the RSI × FixMix interaction, *F*(1,47) = 9.2, *p *= 0.004, $\eta_{\text{p}}^{ 2}$= 0.16, revealed that RTs reduced more with display of the key-specific stimulus in the fixed than in the mixed RSI conditions (24 ms vs. 9 ms, respectively, see Fig. 1). Still, planned comparisons showed that this benefit was significant in both the fixed (i.e., between different subblocks) and mixed (within subblock) conditions, *F*s(1,47) > 22.3, *p*s < 0.001, $\eta_{\text{p}}^{ 2}$s > 0.32. This indication that the benefit of stimulus display in the mixed condition was smaller than in the fixed condition indicates that the S-R channel was no longer entirely open in the mixed condition. This benefit of stimulus display was significant in each of the four Structure by FixMix conditions, *F*s(1,47) > 9.3, *p*s < 0.004, $\eta_{\text{p}}^{ 2}$= 0.17. In the 800-RSI condition, RTs were shorter in the mixed than in the fixed condition (Fig. 1), *F*(1,47) = 4.22, *p *= 0.04, $\eta_{\text{p}}^{ 2}$= 0.08. RTs were not different in the fixed 0-RSI and the mixed 0-RSI conditions, *F*(1,47) = 1.47, *p *= 0.23. In short, displaying a key-specific stimulus immediately following the preceding key press in the 0-RSI condition reduced response time in both fixed and mixed conditions, but the benefit relative to the 800-RSI condition was smaller in the mixed than in the fixed condition.

**Fig. 2:**
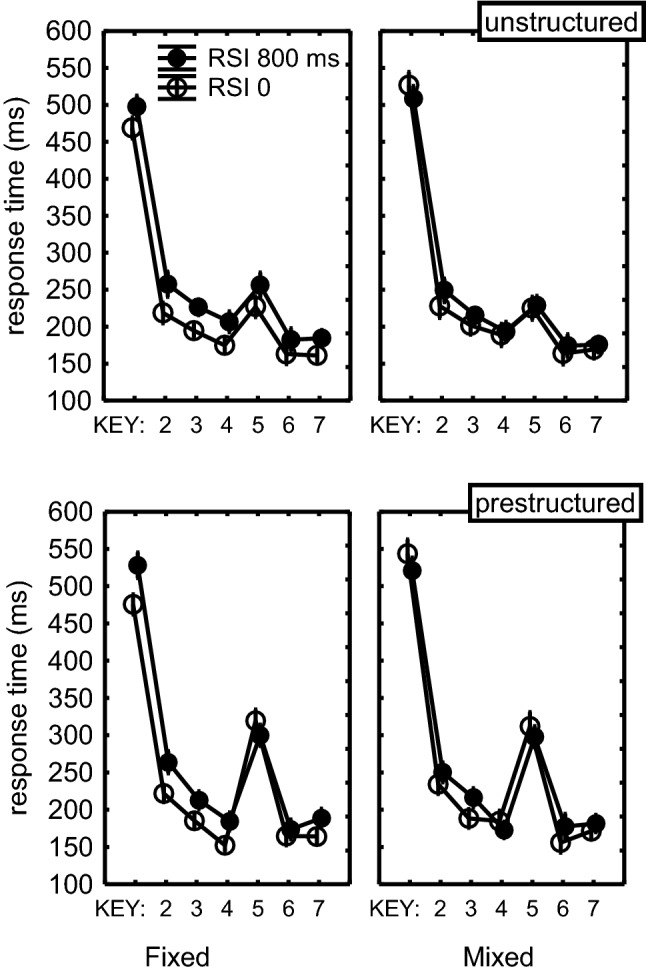
The benefit in Experiment 1 of stimulus display (RSI = 0) in the fixed and mixed conditions, separately for unstructured and prestructured sequences. Only sequences are included with all T_234567_s < 800 ms. Error bars indicate the SEM

The Structure × Key interaction, *F*(5,235) = 11.4, *p *< 0.001, $\eta_{\text{p}}^{ 2}$ = 0.20, along with planned testing of the R_5_ versus R_23467_ × Structure interaction, *F*(1,47) = 38.7, *p *< 0.001, $\eta_{\text{p}}^{ 2}$ = 0.45, confirmed that R_5_ was slowed more in the prestructured than in the unstructured sequence (see Fig. 2). The RSI × Key interaction, *F*(5,235) = 5.1, *p *< 0.001, $\eta_{\text{p}}^{ 2}$ = 0.10, suggested that R_23467_ benefitted more from stimulus display than R_5_. According to the RSI × Structure × Key interaction this change in benefit was different for the unstructured and prestructured sequences, *F*(5,235) = 3.7, *p *< 0.01, $\eta_{\text{p}}^{ 2}$ = 0.07. Planned comparisons of T_5_ versus T_23467_ showed that this difference in benefit among sequential positions reached statistical significance only for the prestructured sequence (Fig. 2), *F*(1,47) = 21.6, *p *< 0.0001, $\eta_{\text{p}}^{ 2}$= 0.31. But in contrast to the prediction of the race assumption, early stimulus display actually showed a 17 ms cost of early stimulus display in R_5_, whereas it was a 20 ms benefit for the remaining responses. Instead, in the unstructured sequence the benefits for R_5_ and R_23467_ did not differ, and these amounted to 20 ms for R_5_ and R_23467_, *F*(1,47) = 0.1, *p* = 0.73. A further planned comparison confirmed that the benefit difference for R_5_ vs. R_23467_ was different for the unstructured and prestructured sequences, *F*(1,47) = 9.9, *p* = 0.003, $\eta_{\text{p}}^{ 2}$ = 0.17.

Finally, a test on just T_23467_ (excluding T_5_) showed that the benefit of stimulus display reduced with sequence position, across unstructured and prestructured sequences: 29 ms, 25 ms, 14 ms, 15 ms, 16 ms, respectively, *F*(4,188) = 3.4, *p* = 0.01, $\eta_{\text{p}}^{ 2}$ = 0.07. This suggests that the reliance on key-specific stimuli reduced with sequential position. In short, the benefit of stimulus display was observed at all positions after R_1_, except at R_5_ in the prestructured sequence, and this benefit reduced with sequential position in prestructured and unstructured sequences.

A 3 (Mixed, Fixed 0-RSI, Fixed 800-RSI) × 2 (Structure) ANOVA on T_1_ showed only that T_1_ was shorter in the fixed 0-RSI condition (474 ms) than in the mixed 800-RSI and the fixed 800-RSI conditions (both: 519 ms), *F*(2,94) = 19.2, *p *< 0.01, $\eta_{\text{p}}^{ 2}$ = 0.29.

Error rates were generally quite low in the test block. A 2 (RSI) × 2 (FixMix) × 2 (Structure) × 6 (Key: R_234567_) repeated measures ANOVA on arcsine transformed error proportions showed main effects of RSI, *F*(1,47) = 9.2, *p* = 0.004, $\eta_{\text{p}}^{ 2}$ = 0.16, and of Key, *F*(5,235) = 3.4, *p* = 0.005, $\eta_{\text{p}}^{ 2}$ = 0.07, implying that more errors were made in the RSI 0 than in the RSI 800 ms condition (2.7 % vs. 3.0 % per key), and that error rate varied between 1.6 % at R_7_ and 3.6 % at R_4_. The RSI × FixMix interaction showed that error rate differed more for RSI 0 and RSI 800 in mixed (2.6 % vs. 3.1 %, resp.) than in fixed conditions (2.7 % vs. 2.9 %), *F*(1,47) = 7.5, *p* = 0.009, $\eta_{\text{p}}^{ 2}$ = 0.14.

#### Awareness

By the end of the practice, phase participants had become reasonably aware of the sequences they had been practicing. Of the 48 participants, 26 (54 %) were able to correctly write down from memory both sequences in terms of the keys they had pressed, 30 (63 %) reproduced the unstructured sequence correctly, and 38 (79 %) reproduced the prestructured sequence correctly. Five of the 48 participants (10 %) were not able to write down either sequence. The notion that awareness was used to anticipate the stimuli that were displayed only after 800 ms was confirmed in that participants with more sequences of which T_2_–T_7_ were below 800 ms (across the fixed 800 and mixed 800-RSI conditions) also wrote down more sequences correctly, *r*(*n* = 48) = 0.28, *p* = 0.05. Extending the above ANOVA for the test RTs with an awareness variable showed a similar RT pattern as in Fig. 1 for the 26 fully aware participants and the remaining 22 less aware participants. This similarity of the RT patterns was confirmed by the RSI × FixMix × Awareness interaction not reaching statistical significance, *F*(1,46) = 2.11, *p* = 0.15. Planned comparisons showed that not only the less aware, but also the fully aware group benefited from early stimulus display in the mixed condition, *F*s(1,46) > 6.89, *p*s < 0.001, $\eta_{\text{p}}^{ 2}$s > 0.14.

### Discussion

The main question in Experiment 1 was whether participants stop using external guidance by isoluminant key-specific stimuli when sequence representations have developed, and whether slow concatenation responses show a larger benefit of these key-specific stimuli than fast execution responses. The results showed the two phenomena needed for testing our predictions. First, not displaying stimuli in the fixed 800-RSI slowed responses by 24 ms relative to the fixed 0-RSI conditions (remember, analyses excluded RTs > 800 ms). This confirmed that with the present amount of practice in the fixed 0-RSI condition participants still benefit from the key-specific stimuli (e.g., Ruitenberg et al., 2014 found a 32 ms slowing after 720 practice trials; also see Verwey, 1999; Verwey et al., 2011). The second phenomenon needed was the development of a relatively slow R_5_ that indicates concatenation. As expected, the slow R_5_ emerged not only in the prestructured sequences (as in Verwey, 1996; Verwey et al., 2014; Verwey et al., 2009; Verwey & Dronkert, 1996), but also developed spontaneously in the unstructured sequences (as in De Kleine & Verwey, 2009; Verwey et al., 2014; Verwey & Dronkers, 2019).

The important mixed RSI condition of the test phase showed that after considerable practice responses were still fastened by the occasional display of key-specific stimuli. However, the smaller RT benefit of stimulus display in the mixed than in the fixed conditions suggests that the S-R channel was closed in part. This benefit reduction is inconsistent with the mandatory processing hypothesis but can be explained by the intentional S-R translation and the fast-sequencing hypotheses. Awareness was limited so according to the intentional S-R translation hypothesis participants did not execute the sequences using explicit sequence knowledge (Tubau & López-Moliner, 2004). According to the fast-sequencing hypothesis, the S-R channel was not yet entirely closed with the present number of practice trials, but that should happen with more extensive practice. So, the question was whether the partial closure of the S-R channel in the mixed condition reflected a strategic adjustment related to the limited likelihood of stimulus display, or insufficient practice to entirely close the S-R channel.

The prediction of the race assumption that displaying stimuli in the mixed RSI condition would have a greater benefit for the concatenation response R_5_ than for the execution responses R_23467_ was not supported. In unstructured sequences, this benefit was the same for the slower concatenation and the faster execution responses. In the prestructured sequences, early stimulus display did not fasten but, instead, slowed R_5_. This was in line with S_5_ processing being slowed by concatenation. It also confirms the earlier indications that concatenation in prestructured sequences differs from that in unstructured sequences (Jouen et al., 2013; Ruitenberg et al., 2015).

Two unexpected observations were that both the beneficial effect on RT of stimulus display after 0-RSI, and the proportion of responses on which participants waited for the stimulus after 800 ms reduced with sequence position. These observations indicate that the contribution of stimuli reduced toward the end of the sequences.

## Experiment 2

The second experiment tested whether the smaller benefit of stimulus display in the mixed than in the fixed test condition of Experiment 1 was caused by a slowly developing dominance of sequencing systems over the S–R translation system, which would fit the fast-sequencing hypothesis, or whether participants willfully closed the S–R channel to an extent associated with the stimulus display probability, which would be in line with the intentional S–R translation hypothesis.

Experiment 2 was like Experiment 1 except that participants alternated between practice and test blocks. This allowed exploring how the benefit of the occasional early key-specific stimulus in the mixed and fixed conditions develops with practice. This time, all sequences were unstructured, and I counted on the spontaneous development of a concatenation response at the 5^th^ position to test the prediction of the race assumption that the slow concatenation response R_5_ benefits more from key-specific stimuli than the execution responses R_23467_.

### Method

#### Participants

Twenty-four undergraduate students took part (age range 18–26 years, mean age 20.6, 12 women, 1 lefthander) in exchange for course credits. This number was deemed appropriate as the lowest observed effect size of stimulus display in each of the Structure by FixMix conditions in Experiment 1 amounted to $\eta_{\text{p}}^{ 2}$= 0.17. With this $\eta_{\text{p}}^{ 2}$ and the typical other parameters (*α *= 0.05, 1−*β* = 0.85), GPower indicated for a repeated measures design, an a priori sample size of 14 participants. This was increased to 24 given the larger variance that I expected in the early test block, and the need to fully counterbalance across finger positions per sequence element and test conditions. None of the participants was color blind. The study had been approved by the ethics committee of Faculty of Behavioral Sciences of the University of Twente, and all participants signed an informed consent.

#### Apparatus

Stimulus presentation, timing, apparatus, and data collection were as described with Experiment 1, except that this time the experiment ran on a standard Windows XP PC of which unnecessary Windows services were shut down.

#### Task and procedure

In Experiment 2, the practice phase occurred in Blocks 1, 3, and 5, each of which including 90 trials for each of the two unstructured 7-key sequences. By the time participants started the last test (Block 6), they had practiced each sequence for 270 trials (which is almost half the 450 trials in Experiment 1). The test phase consisted of Blocks 2, 4, and 6. Each block included the fixed 0-RSI, fixed 800-RSI, and the mixed RSI conditions in three subblocks, the order of which was counterbalanced across the participants. Each test block included 40 trials of each sequence. The awareness questionnaire was filled in after Block 5, before the last test block. The duration of the experiment was again about two and a half hours.

### Results

#### Practice blocks

Response times in the practice blocks were subjected to a 3 (Block: 1, 3, 5) × 7 (Key) ANOVA. The results showed the typical effects of practice: main effects of Key, *F*(6,138) = 52.3, *p *< 0.001, $\eta_{\text{p}}^{ 2}$ = 0.69, and Block, *F*(2,46) = 359.7, *p *< 0.001, $\eta_{\text{p}}^{ 2}$= 0.94, and a Block × Key interaction, *F*(12,276) = 23.3, *p *< 0.001, $\eta_{\text{p}}^{ 2}$ = 0.50, that indicated the typical finding that practice fastens R_23467_ more than R_1_ and R_5_. Planned comparisons showed a significant R_5_ vs. R_23467_ by Block interaction, *F*(2,46) = 6.37, *p* = 0.003, $\eta_{\text{p}}^{ 2}$ = 0.29, confirming that R_5_ reduced less with practice than R_23467_. Indeed, R_5_ was 8 ms slower than R_23467_ in Block 1, *F*(1,23) = 0.92, *p* = 0.35, 49 ms slower in Block 3, and 43 ms slower in Block 5, *F*s(1,23) > 9.60, *p*s < 0.006, $\eta_{\text{p}}^{ 2}$< 0.29.

The same ANOVA on arcsine transformed error proportions showed an almost linear, but small, error rate increase across practice blocks, from 1.5 % per key in Block 1 to 2.4 % per key in Block 5, *F*(2,46) = 11.8, *p *< 0.001, $\eta_{\text{p}}^{ 2}$= 0.34. Error rate differed across sequence positions, varying between 1.1 % per key for Key 1, and 2.5 % for Key 6, *F*(6,138) = 2.9, *p *= 0.01, $\eta_{\text{p}}^{ 2}$= 0.11. The Block by Key interaction indicated that especially Key 6 in Block 5 involved a high error percentage, 4.0 %, *F*(12,276) = 2.3, *p *= 0.009, $\eta_{\text{p}}^{ 2}$= 0.09.

#### Test blocks

First, a 3 (Block: 2, 4, 6) × 2 (RSI: 0 vs. 800 ms) × 2 (FixMix: Fixed RSI vs. Mixed RSI) × 6 (Key: 2–7) repeated measures ANOVA was performed across all participants on arcsine transformed proportions of RTs over 800 ms in correct sequences. It showed that the proportions of RTs over 800 ms reduced across Block, *F*(2,46) = 21.5, *p *< 0.001, $\eta_{\text{p}}^{ 2}$= 0.48. These proportions were larger with RSI 800 than with RSI 0, 15 % vs 1 %, resp., indicating that in the RSI 800 condition participants sometimes waited for the stimulus, *F*(1,23) = 19.6, *p *< 0.001, $\eta_{\text{p}}^{ 2}$= 0.46. The proportions of RTs over 800 ms reduced similarly across the test blocks in the fixed 800-RSI and mixed 800-RSI conditions: In the fixed 800-RSI condition from 32 % in Block 2, to 11 % in Block 4, and 4 % in Block 6. In the mixed 800-RSI condition, these percentages amounted 27 %, 10 %, and 8 %, respectively.

According to a Key main effect, *F*(5,115) = 11.5, *p *< 0.001, $\eta_{\text{p}}^{ 2}$= 0.33, and a Key by RSI interaction, *F*(5,115) = 11.6, *p *< 0.001, $\eta_{\text{p}}^{ 2}$= 0.34, the proportion of RTs over 800 ms in the 800 ms RSI conditions reduced across successive keys in the mixed and fixed conditions (from R_2_ to R_7_: 19 %, 19 %, 16 %, 17 %, 13 %, 6 %), while they remained consistently low for all sequence positions in the RSI 0 conditions (all below 1.5 %). According to a Key × RSI × Block interaction, *F*(10,230) = 3.8, *p *< 0.001, $\eta_{\text{p}}^{ 2}$ = 0.14, this reduction with sequence position became less pronounced in later blocks because the proportions of responses over 800 ms at the earlier positions reduced across blocks. For instance, the proportion RTs > 800 ms at R_2_ reduced from 35 % in Block 2 to 12 % in Block 4, and to 10 % in Block 6. This reduction with practice was smaller at later sequential positions.

The RT analysis involved all responses except R_1_ in errorless sequences with RTs below 800 ms. So, the ANOVA involved responses from sequences in which all responses had been executed before display of the key-specific stimulus. Five participants were excluded from this analysis because missing data for some sequence positions indicated that at those positions they had always waited for stimulus display in the fixed 800-RSI and/or mixed 800-RSI conditions in Blocks 2 and 4 (this did not happen for any participant in Block 6). These RTs were, again, analyzed with a 3 (Block) × 2 (RSI) × 2 (FixMix) × 6 (Key 2–7) repeated measures ANOVA.

**Fig. 3:**
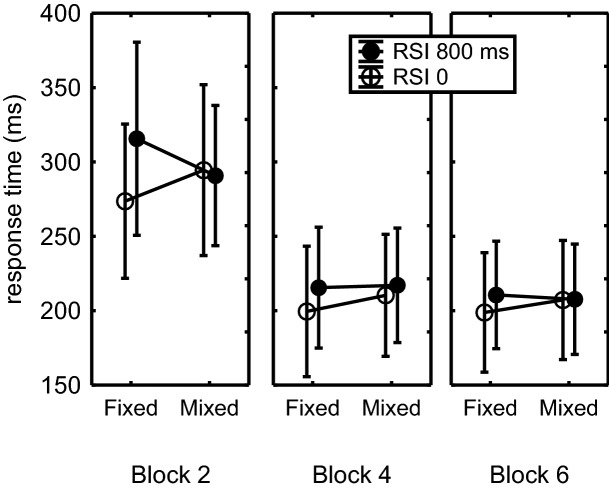
Mean response times across T_2_–T_7_ as a function of fixed RSI versus mixed RSI in Blocks 2, 4, and 6 of Experiment 2. Only sequences are included in which all RTs were below 800 ms. Error bars indicate the SEM (like in Fig. 2 SEM values were quite large because they also include the differences across T_2_–T_7_, cf. Fig. 4)

This ANOVA showed significant main effects of Block, *F*(2,36) = 59.7, *p *< 0.001, $\eta_{\text{p}}^{ 2}$ = 0.77, RSI, *F*(1,18) = 13.0, *p* = 0.002, $\eta_{\text{p}}^{ 2}$ = 0.42, and Key, *F*(5,90) = 14.4, *p *< 0.001, *η*_p_^2^ = 0.44. The RSI × FixMix interaction showed that the effect of RSI—indicating the benefit of a key-specific stimulus—was greater in the fixed than in the mixed conditions, *F*(1,18) = 9.5, *p* = 0.006, $\eta_{\text{p}}^{ 2}$ = 0.35. According to a Block × RSI × FixMix interaction, *F*(2,36) = 4.2, *p* = 0.02, $\eta_{\text{p}}^{ 2}$ = 0.19, this RSI effect reduced with practice (Fig. 3). Planned comparisons confirmed that the benefit from displaying the key-specific stimulus (i.e., RSI 0 vs. RSI 800) was larger in the fixed than in the mixed condition in Block 2, *F*(1,18) = 8.2, *p* = 0.01, $\eta_{\text{p}}^{ 2}$ = 0.31, but not so in Blocks 4 and 6, *F*s(1,18) < 2.4, *p*s > 0.14. In other words, the occasional display of a key-specific stimulus had a beneficial effect only in Block 2 if it was always displayed (i.e., in the fixed 0 condition), but not when the stimulus appeared occasionally and unpredictably (i.e., in the mixed 0 condition).

In the fixed RSI conditions displaying the stimulus at RSI 0 instead of after 800 ms reduced RTs when tested across Blocks 2, 4, and 6, *F*(1,18) = 15.4, *p *= 0.001, $\eta_{\text{p}}^{ 2}$= 0.46. This benefit reached significance also when tested separately in Blocks 2 and 4 (42 and 16 ms, respectively), *F*s(1,18) > 8.2, *p*s ≤ 0.01, $\eta_{\text{p}}^{ 2}$s > 0.31, but not in Block 6 (12 ms), *F*(1,18) = 2.7, *p *= 0.12 (Fig. 3). Conversely, in the mixed condition, there was no benefit of immediate stimulus display across all three test blocks (− 4 ms, 7 ms, 0 ms, respectively), *F*(1,18) = 0.1, *p* = 0.76. Across all three blocks, execution was faster in the fixed 0-RSI than in the mixed 0-RSI condition, *F*(1,18) = 4.6, *p* = 0.046, $\eta_{\text{p}}^{ 2}$ = 0.20. Yet, despite the significant RSI × FixMix interaction in Block 2, *F*(1,18) = 8.24, *p* = 0.01, $\eta_{\text{p}}^{ 2}$ = 0.31 (Fig. 3), planned comparison showed no significant difference between the fixed and mixed 0-RSI conditions, *F*(1,18) = 1.6, *p* = 0.22, and neither between the fixed and mixed 800-RSI conditions, *F*(1,18) = 2.2, *p* = 0.15.

**Fig. 4:**
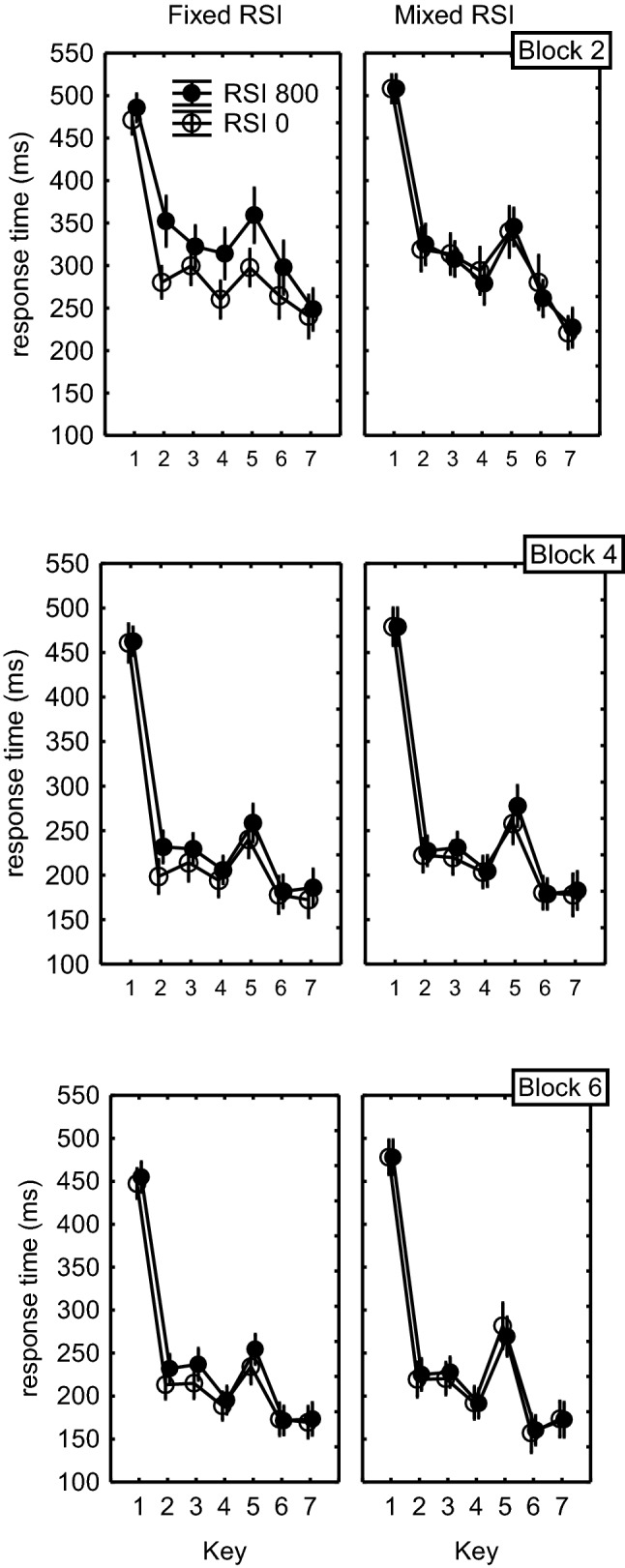
Response times as a function of fixed-mixed condition and key position in Blocks 2, 4, and 6 of Experiment 2. Error bars indicate the SEM

The ANOVA further showed that Key interacted with Block, *F*(10,180) = 3.2, *p *< 0.001, $\eta_{\text{p}}^{ 2}$= 0.15, indicating that the effect of practice was different at the various sequential positions (Fig. 4). The Key × RSI, *F*(5,90) = 3.1, *p* = 0.01, $\eta_{\text{p}}^{ 2}$ = 0.15, Key × FixMix, *F*(5,90)= 4.6, *p *< 0.001, $\eta_{\text{p}}^{ 2}$= 0.20, and Key × RSI × FixMix interactions, *F*(5,90)= 2.4, *p*= 0.046, $\eta_{\text{p}}^{ 2}$= 0.12, revealed that across the three test blocks the benefit of stimulus display in the fixed conditions reduced with sequential position (for T_2_–T_7_: 41 ms, 20 ms, 24 ms, 33 ms, 12 ms, 9 ms, respectively, see Fig. 4). Of course, such a benefit reduction with sequence position was not observed in the mixed condition. There it ranged between 5 ms at T_2_ and 3 ms at T_7_. This difference between the fixed and mixed conditions was supported by a Block × RSI × FixMix × Key interaction, *F*(10,180)= 2.4, *p*= 0.01, $\eta_{\text{p}}^{ 2}$= 0.12.

Planned comparisons of the stimulus display benefit in the fixed conditions across Blocks 2, 4, and 6 were in line with the predicted benefit difference of early stimulus display for R_5_ (33 ms) and R_23467_ (21 ms), but this did not reach statistical significance *F*(1,18)= 2.7, *p*= 0.12. Such a difference was not observed in the individual test blocks either, *F*s(1,18) < 2.5, *p*s > 0.13. Of course, in the mixed condition the benefit of stimulus display did not differ for different responses because that condition did not show an effect of early stimulus display any way.

Taken together, stimulus display in the fixed 0-RSI conditions showed a benefit that reduced with sequential position and that was not significantly different for R_5_ and R_23467_. This effect diminished in successive blocks. The mixed condition did not show any significant benefit of stimulus display.

A 3 (Block) × 2 (RSI) × 2 (FixMix) repeated measures ANOVA on T_1_, including only the same errorless sequences with all RTs below 800 ms, showed main effects of Block, *F*(2,46)= 5.7, *p*= 0.006, $\eta_{\text{p}}^{ 2}$= 0.20, indicating that T_1_ reduced somewhat across Blocks 2, 4, and 6 (484, 468, 465 ms), and of FixMix indicating faster sequence initiation in fixed than in mixed, 463 vs. 481 ms, *F*(1,23)= 14.6, *p *< 0.001, $\eta_{\text{p}}^{ 2}$= 0.39.

The analysis of arcsine transformed errors involved a 3 (Block) × 2 (RSI) × 2 (FixMix) × 7 (Key) repeated measures ANOVA. It showed that more errors were made in the 800-RSI than in the 0-RSI conditions, 2.4 % vs. 2.0 % per key, *F*(1,23)= 24.9, *p *< 0.001, $\eta_{\text{p}}^{ 2}$= 0.52, and that error rate per key was lowest at R_1_ and R_7_ (1.1 % and 1.6 %) and varied between 1.9 % and 2.9 % for R_2_–R_6_, *F*(6,138)= 4.8, *p*= 0.001, $\eta_{\text{p}}^{ 2}$= 0.17. Interactions showed that error rate was especially low in Block 2 for RSI 0, 1.5 % per key, while for the remaining 0 and 800-RSI conditions in Blocks 2, 4, and 6 it ranged between 2.0 % and 2.6 % per key, *F*(2,46)= 7.7, *p*= 0.001, $\eta_{\text{p}}^{ 2}$= 0.25. Error rate was equal for fixed 0 and fixed 800-RSI condition, both 2.3 %, which was higher than that in mixed 800-RSI, 2.5 %, and lower than that in mixed RSI 0, 1.7 %, *F*(1,23)= 6.4, *p*= 0.02, $\eta_{\text{p}}^{ 2}$= 0.22.

#### Awareness

At the end of the last practice block, Block 5, participants had obtained almost full awareness of the sequences they had been practicing. Of the 24 participants, 22 (92 %) were able to correctly write down both sequences from memory. Two participants made an error in one sequence, one of which involved just a single sequence element in an otherwise correct sequence.

### Discussion

The results of Experiment 2 showed that in the fixed RSI conditions of the three test blocks (i.e., Blocks 2, 4, and 6), the benefit of stimulus display was substantial in the first test block, was smaller in the second test block, and was smallest and no longer significant in the third test block. This reduction of the benefit of stimulus display occurred with much less practice than in Experiment 1. Moreover, stimulus display did not show any benefit in the mixed test conditions. These findings are inconsistent with the fast-sequencing hypothesis which does not predict full elimination of the benefit of stimulus display with even less practice than in Experiment 1, and that this would be so different in the fixed and mixed conditions. Instead, these results can be explained by the intentional S-R translation hypothesis in that participants ignored isoluminant key-specific stimuli depending on the test condition. Additional support for this hypothesis comes from the finding that participants had exceptionally high awareness of the sequences. This may have allowed them to entirely rely on explicit sequence knowledge, just like in the serial RT task (Tubau & López-Moliner, 2004). Still, we cannot exclude that full awareness gave participants the confidence to rely entirely on their implicit sequence knowledge and ignore the key-specific stimuli.

Why was awareness so much higher in Experiment 2 than in Experiment 1? Such high awareness is uncommon for DSP experiments after the typical 500 practice trials per sequence, and probably even less common after the present 270 practice trials (for an overview, see Verwey et al., 2016, also see Experiment 1). Most likely, executing sequences in the mixed 800-RSI and fixed 800-RSI conditions of the first test block stimulated participants to develop explicit sequence knowledge because hypotheses on element order could be tested easily (like in the serial RT task, Frensch & Rünger, 2003; Rünger & Frensch, 2008). Here, this involved only delaying the response.

Like the unstructured sequences of Experiment 1, unstructured sequences in Experiment 2 showed the spontaneous development with practice of a relatively slow R_5_. Still, the data again did not show that the benefit of early stimulus display was larger for the slow concatenation response R_5_ than for the fast execution responses R_23467_. This again refutes C-SMB’s race assumption.

The test blocks of Experiment 2 replicated the observation in Experiment 1 of a monotonically reducing benefit of key-specific stimuli toward the end of the sequences, from 41 ms at R_2_ to 9 ms at R_7_. This was observed also as a reducing proportion of stimulus-dependent responses (i.e., with RTs over 800 ms) in the fixed 800-RSI and the mixed 800-RSI conditions.

## General discussion

In the present study, I tested three hypotheses. These involved (a) that after practice key-specific stimuli are no longer used, (b) that participants can choose whether these stimuli are being used, and (c) that stimuli continue to be used irrespective of amount of practice. I tested these hypotheses by assessing for the situation that these stimuli do not capture attention whether the so-called S-R translation channel is closed when stimuli are displayed only occasionally. Experiment 1 examined the benefit of these occasionally displayed stimuli after substantial practice, while Experiment 2 explored how this benefit develops across practice. I further tested the prediction of C-SMB’s race assumption that a potential benefit of key-specific stimuli should be larger for slow concatenation responses than for fast execution responses.

### Flexible use of the key-specific stimuli

The results supported the intentional S-R translation hypothesis which states that participants can choose whether they use key-specific stimuli. Experiment 1 already showed that after extensive practice the use of these stimuli is attenuated. Then, Experiment 2 showed that this reducing attention to the key-specific stimuli was not caused by practice per se in that participants appeared able to entirely refrain from processing the stimuli with limited practice too.

The reason that participants in Experiment 2 chose to ignore key-specific stimuli so early in practice was probably because they quickly developed full explicit sequence knowledge. The high awareness in Experiment 2 was exceptional for DSP tasks and was most likely caused by performing so early in practice in the 800-RSI conditions. This does not necessarily mean that sequence execution in Experiment 2 relied solely on explicit sequence knowledge (as may have happened in the serial RT task, Tubau & López-Moliner, 2004). As explicit sequence knowledge probably allows for low execution rates only (Verwey, 2015), full awareness of the sequences may have convinced the participants that they could rely on their implicit sequence knowledge.

The finding that the key-specific stimuli were still used after extended practice in Experiment 1 suggests that keeping the S-R translation channel open demands little cognitive effort for the present situation with spatially compatible responses (Hommel, Müsseler, Aschersleben, & Prinz, 2001; Kornblum, Hasbroucq, & Osman, 1990). Attending to the area where the stimuli are displayed may have sufficed for automatically priming the spatially corresponding response, even in the situation with isoluminant stimuli (Rizzolatti et al., 1987; Van der Lubbe et al., 2012).

Interestingly, in both experiments the beneficial effect of the occasionally displayed stimuli in the mixed conditions gradually reduced with sequence position. This was indicated by a reducing RT benefit, and by a reducing number of times participants waited for stimulus display toward the end of the sequences. These findings might reflect that disengaging attention from the stimulus display area after S_1_ identification takes some time, possibly because cognitive processing resources were allocated to sequence execution (Belopolsky et al., 2010; Theeuwes, 2010). It is possible also that this effect is not attentional, and that the dominance of sequence representations increases with sequential position due to an accumulation of activation (MacKay, 1982; Verwey, 1994; Verwey & Abrahamse, 2012).

### Revising the race assumption

The race assumption predicts that the benefit of early stimulus display in the mixed condition should be greater for the slow concatenation response R_5_ than for the faster execution responses R_23467_ because S-R translation more often wins the race with the competing sequencing systems when triggering R_5_. Neither Experiment 1 nor Experiment 2 corroborated this. For unstructured sequences, the beneficial effect of stimulus display appeared the same for all non-initial responses. This may reflect that key-specific stimuli prime the associated responses, but that those responses are executed only when they are confirmed by a relevant sequence representation (cf. Kornblum et al., 1990).

In the prestructured sequences of Experiment 1, concatenation response R_5_ was slowed by S_5_ display, instead of fastened. First of all, this supports that concatenation in prestructured sequences differs from that in unstructured sequences (Ruitenberg et al., 2015). Given the indications that only in prestructured sequences removing the pause induces a concatenation process that requires cognitive processes (Jouen et al., 2013), this finding suggests that processing S_5_ slows this concatenation process, which it does not with concatenation in unstructured sequences.

## Conclusions

The results of the present study (a) corroborate that participants can intentionally ignore isoluminant key-specific stimuli in familiar, discrete keying sequences. Experiment 1 suggests that participants tend to continue using these stimuli when displayed. Experiment 2 suggests that processing key-specific stimuli is a strategic decision that is based on the participants’ insight that they possess well-developed (explicit or implicit) sequence representations. (b) In contrast with the race hypothesis, after practice S–R translation seems to prime responses and execution requires confirmation by a sequence representation. (c) The finding that concatenation is more susceptible to concurrent stimulus processing in prestructured than in unstructured sequences suggests that concatenation of segments that used to be separated in time requires cognitive processing resources that can be interfered with by stimulus identification. Finally, (d) Experiment 2 suggests that the fixed and mixed 800-RSI conditions provide an efficient procedure for developing explicit sequence knowledge in early practice, most likely because participants can test their hypothesis on an upcoming response simply by delaying that response.

## Footnotes

1 Non-aging intervals are intervals with a decreasing probability of ending as time progresses (Gottsdanker, Perkins, & Aftab, 1986). I computed the non-aging interval in a programming loop starting at 300 ms. The loop each time added 5 ms and had a 1 % chance to stop until 6,000 ms was reached and it stopped anyway. Then, the computer waited for the computed interval.
